# Modification of Experimental Design and Statistical Method for Mapping Imprinted QTLs Based on Immortalized F_2_ Population

**DOI:** 10.3389/fgene.2020.589047

**Published:** 2020-11-20

**Authors:** Kehui Zheng, Jiqiang Yan, Jiacong Deng, Weiren Wu, Yongxian Wen

**Affiliations:** ^1^College of Life Sciences, Fujian Agriculture and Forestry University, Fuzhou, China; ^2^College of Computer and Information Sciences, Fujian Agriculture and Forestry University, Fuzhou, China; ^3^School of Ocean and Biochemical Engineering, Fuqing Branch of Fujian Normal University, Fuzhou, China; ^4^Fujian Provincial Key Laboratory of Crop Breeding by Design, Fujian Agriculture and Forestry University, Fuzhou, China; ^5^Key Laboratory of Genetics, Breeding and Multiple Utilization of Crops, Ministry of Education, Fujian Agriculture and Forestry University, Fuzhou, China

**Keywords:** genomic imprinting, imprinted quantitative trait loci, point mapping, composite point mapping, immortalized F_2_ population

## Abstract

Genomic imprinting is an epigenetic phenomenon, which plays important roles in the growth and development of animals and plants. Immortalized F_2_ (imF_2_) populations generated by random cross between recombinant inbred (RI) or doubled haploid (DH) lines have been proved to have significant advantages for mapping imprinted quantitative trait loci (iQTLs), and statistical methods for this purpose have been proposed. In this paper, we propose a special type of imF_2_ population (R-imF_2_) for iQTL mapping, which is developed by random reciprocal cross between RI/DH lines. We also propose two modified iQTL mapping methods: two-step point mapping (PM-2) and two-step composite point mapping (CPM-2). Simulation studies indicated that: (i) R-imF_2_ cannot improve the results of iQTL mapping, but the experimental design can probably reduce the workload of population construction; (ii) PM-2 can increase the precision of estimating the position and effects of a single iQTL; and (iii) CPM-2 can precisely map not only iQTLs, but also non-imprinted QTLs. The modified experimental design and statistical methods will facilitate and promote the study of iQTL mapping.

## Introduction

Genomic imprinting is a phenomenon found in animal and plant, in which two alleles of a gene show unequal expression depending on their parental origins. Genes involved in such phenomenon are called imprinted genes. Many imprinted genes have been found in animal and human (Nolan et al., 2001; Morison et al., 2005; Long and Cai, 2007; Babak et al., 2008; Hagan et al., 2009; Girardot et al., 2013; Pembrey et al., 2014; Hur et al., 2016; Jiang et al., 2017; Mackay and Temple, 2017). In plant, the first imprinted gene, which is involved in the coloration of maize kernel endosperm, was discovered as early as about half centuries ago (Kermicle, 1970). Compared with those in animal, however, the imprinted genes identified in plant so far are still very limited, of which most are found from *Arabidopsis*, rice and maize (Danilevskaya et al., 2003; Haun et al., 2007; Luo et al., 2009; Bauer and Fischer, 2011; Raissig et al., 2011; Zhang et al., 2011; Ikeda, 2012; Pei et al., 2019).

It has been found that many quantitative traits are affected by genomic imprinting (Spencer, 2002; Croteau and Croteau, 2004; Sandovici et al., 2005; Santure and Spencer, 2011; Wang et al., 2012). The quantitative trait loci (QTLs) showing imprinting effect are called imprinted QTL (iQTL). A number of different experimental designs and corresponding statistical methods have been proposed for mapping iQTLs (Knott et al., 1998; de Koning et al., 2000; Pratt et al., 2000; Strauch et al., 2000; Hanson et al., 2001; Haghighi and Hodge, 2002; Shete and Amos, 2002; Shete et al., 2003; Knapp and Strauch, 2004; Mantey et al., 2005; Cui et al., 2006, 2007, 2008, Cui, 2007; Liu et al., 2007; Li et al., 2008, 2012a; Hager et al., 2008; Yang et al., 2010; Zhou et al., 2012; Karami et al., 2019). F_2_ (outbred or inbred) and BC_1_ populations are usually used for iQTL mapping (Haley et al., 1994; de Koning et al., 2000; Cui et al., 2006; Li et al., 2012a), but they have obvious shortcomings, such as relatively low power in iQTL detection, low accuracy in estimating the positions and effects of iQTLs, inability of permanent preservation of the population, and unrepeatability. Besides, determination of the parental origins of alleles is also difficult or problematic under the F_2_ and BC_1_ designs (Wu et al., 2002; Cui et al., 2006; Wolf et al., 2008; Lawson et al., 2013). In addition, the imprinting effect cannot be separated from the maternal effect in the BC_1_ design.

Wen and Wu (2014) proposed statistical methods for iQTL mapping using an immortalized F_2_ (abbreviated as imF_2_) population generated from random crosses between recombinant inbred (RI) lines or doubled haploid (DH) lines. Compared with the previous designs, the imF_2_ design has significant advantages for iQTL mapping. First, the parental origins of marker alleles in each imF_2_ line can be exactly known from the cross. Second, analysis based on imF_2_ lines can reduce environmental error so as to increase the statistical power of iQTL mapping. Third, a very large imF_2_ population can be produced without increasing the cost of molecular marker assay. However, there are also shortcomings in the experimental design and mapping methods proposed by Wen and Wu (2014). In the experimental design, the work of constructing an imF_2_ population is laborious. In the statistical methods, iQTL mapping is performed only by testing the imprinting effect without making use of the information of additive effect and dominance effect. This may reduce the precision of iQTL mapping.

In this paper, to overcome the above shortcomings, we propose a modified imF_2_ design and modified statistical methods for iQTL mapping based on the work of Wen and Wu (2014). We demonstrate by simulation studies that the modified methods can map both iQTLs and non-imprinted QTLs (niQTLs) simultaneously as well as improve the accuracies of estimation of the positions and effects of iQTLs. In addition, the modified design can potentially reduce the workload in the construction of the imF_2_ population.

## Theory

### Modification of Experimental Design

Suppose there is a DH or RI population derived from a cross between two pure lines, P_1_ and P_2_. The experimental design proposed by Wen and Wu (2014) for iQTL mapping is to develop an imF_2_ population by randomly crossing DH or RI lines, namely, Line *i* × Line *j* (*i*, *j* = 1, 2, 3, …; *i* ≠ *j*). Consider a QTL with two alleles, *Q*_1_ and *Q*_2_. The two alleles can form four genotypes: *Q*_1_*Q*_1_, *Q*_1_*Q*_2_, *Q*_2_*Q*_1_, and *Q*_2_*Q*_2_, with one allele (say, *Q*_1_) from the male gamete and the other (*Q*_2_) from the female gamete in each genotype. Let *a*, *d* and *i* be the additive effect, dominance effect and imprinting effect of the QTL, respectively. Thus, in an imF_2_ population, the single-QTL model would be (Wen and Wu, 2014):

(1) $$y_{j}=\mu +a\,x_{j}+d\,z_{j}+i\,t_{j}+\varepsilon_{j}$$

where *y*_*j*_ is the trait value of the *j*^th^ combination (or hybrid line); μ is the population mean; *x*_*j*_, *z*_*j*_ and *t*_*j*_ are dummy variables taking values depending on the QTL genotype in the *j*^th^ combination (Table 1); and ε_*j*_ is residual error following a normal distribution N(0,σ)2.

**TABLE 1:** Values of dummy variables indicating the QTL genotype in Eq. (1).

**QTL genotype (♀/♂)**	***x*_*j*_**	***z*_*j*_**	***t*_*j*_**
*Q*_1_/*Q*_1_	1	0	0
*Q*_1_/*Q*_2_	0	1	1
*Q*_2_/*Q*_1_	0	1	−1
*Q*_2_/*Q*_2_	−1	0	0

In the above design, the cross in each combination is “unidirectional,” namely, one line is used as female parent and the other as male parent. However, there can be an alternative genetic mating design, in which reciprocal crosses are performed for every combination, namely, Line *i* × Line *j* (positive cross, PC) and Line *j* × Line *i* (negative cross, NC; *i*, *j* = 1, 2, 3, …; *i* < *j*). This modified experimental design generates a special imF_2_ population. We call it reciprocal-cross imF_2_ (R-imF_2_) population. For distinction, we shall call the usual imF_2_ population as unidirectional-cross imF_2_ (U-imF_2_) population. Genetically, Eq. (1) is still applicable to R-imF_2_. Therefore, the iQTL mapping methods for U-imF_2_ are also applicable to R-imF_2_.

### Modification of Point Mapping Method

Suppose the parental DH or RI population has been genotyped and therefore a genetic map has been constructed. Thus, the genotypes of imF_2_ lines can be deduced from the parental DH or RI lines and the genetic map can be used for iQTL mapping. Suppose the genetic map is of ultrahigh density so that the markers can well represent the whole genome. Thus, iQTLs can be mapped by testing every marker throughout the genome. We call this approach point mapping (PM; Wen and Wu, 2014).

Suppose the size (total number of hybrid lines) of the imF_2_ population is 2*n* (for R-imF_2_ population, there are *n* PC and *n* NC hybrid lines, respectively). Let RSS_0_, RSS_1_ and RSS_2_ be the minimum residual sum of squares calculated based on Eq. (1) under the hypotheses *H*_0_: *a* = *d* = *i* = 0, *H*_1_: *i* = 0 but not *a* = *d* = 0, and *H*_2_: not *a* = *d* = *i* = 0, respectively. Thus, two approximate log-likelihood ratio tests can be performed as below:

(2) $${\text{LOD}}_{1}=n\,\left[l\,o\,g_{10}\,\left(R\,S\,S_{1}\right)-l\,o\,g_{10}\,\left(R\,S\,S_{2}\right)\right]$$

and

(3) $${\text{LOD}}_{2}=n\,\left[l\,o\,g_{10}\,\left(R\,S\,S_{0}\right)-l\,o\,g_{10}\,\left(R\,S\,S_{2}\right)\right]$$

The PM method proposed by Wen and Wu (2014) maps iQTLs by checking the imprinting effect of every marker in the genome using Eq. (2). The LOD_1_ significance threshold is estimated by permutation tests (Churchill and Doerge, 1994). A genomic region covered by a LOD_1_ peak exceeding the threshold is thought to harbor an iQTL and the highest point of the peak is the most probable position of the iQTL. Obviously, this is a one-step method (denoted as PM-1), in which an iQTL is mapped based on its imprinting effect only.

The modified PM method proposed here is a two-step method (denoted as PM-2). The first step is QTL mapping, namely, to map QTLs (including imprinted and non-imprinted) by testing every marker in the genome using Eq. (3). Similarly, the LOD_2_ significance threshold used in this step can be estimated by permutation tests. The second step is iQTL identification, namely, to identify iQTLs among the mapped QTLs by checking the imprinting effect of each QTL using Eq. (2). A QTL is taken as an iQTL if its imprinting effect is significant. Otherwise, it is taken as a usual niQTL. The LOD_1_ significance threshold used in the second step can also be estimated by permutation tests, but the tests are performed only on the mapped QTLs rather than on every marker in the genome.

### Modification of Composite Point Mapping Method

The PM method can be extended to composite point mapping (CPM) by adding some markers as cofactors into Eq. (1), namely (Wen and Wu, 2014):

(4) $$y_{j}=\mu +a\,x_{j}+d\,z_{j}+i\,t_{j}+\sum_{k_{1}=1}^{m_{1}}a_{k_{1}}^{*}\,x_{k_{1}\,j}^{*}+\sum_{k_{2}=1}^{m_{2}}d_{k_{2}}^{*}\,z_{k_{2}\,j}^{*}+\sum_{k_{3}=1}^{m_{3}}i_{k_{3}}^{*}\,t_{k_{3}\,j}^{*}+\varepsilon_{j}$$

where *m*_*1*_, *m*_*2*_, and *m*_*3*_, $a_{k_{1}}^{*}$, $d_{k_{2}}^{*}$, and $i_{k_{3}}^{*}$, and $x_{k_{1}\,j}^{*}$, $z_{k_{2}\,j}^{*}$, and $t_{k_{3}\,j}^{*}$ are the numbers, effects and corresponding dummy variables of additive, dominance and imprinting cofactors, respectively; other symbols are the same as those in Eq. (1). Cofactors can be selected by stepwise regression. Note that the three effects of a marker are orthogonal or independent to each other, among which only the significant ones are selected by the stepwise regression. Therefore, the markers selected as cofactors based on different effects can be different (Zeng, 1994). The CPM method proposed by Wen and Wu (2014) is a one-step method, corresponding to PM-1. Similarly, there can be an alternative two-step CPM method (CPM-2). CPM-1 and CPM-2 have a similar procedure to that of PM-1 and PM-2, respectively. The only difference of CPM from PM is that the RSS_0_, RSS_1_ and RSS_2_ in Eqs. (2 and 3) are calculated based on Eq. (4) rather than on Eq. (1) under the corresponding hypotheses (*H*_0_, *H*_1_, and *H*_2_).

### Simulation Studies

To examine the feasibility and efficiency of the modified imF_2_ design (R-imF_2_) and the modified statistical methods (PM-2 and CPM-2) for iQTL mapping in comparison with the previous design (U-imF_2_) and methods (PM-1 and CPM-1), two simulation studies were conducted. The first study was to compare the performances of R-imF_2_ and U-imF_2_, and of PM-1 and PM-2 in the mapping of a single iQTL; the second study was to compare the performances of CPM-1 and CPM-2 as well as PM-1 and PM-2 in the mapping of multiple iQTLs. The programs for PM and CPM were written in Visual Basic 6.0^1^.

### Simulation Study I

In this simulation study, we assumed that an iQTL was located at the middle of a chromosome, which was 100 cM in length and had one marker every cM. The iQTL segregated in a DH population of 100 lines, from which a U-imF_2_ or R-imF_2_ population comprising 800 hybrid lines was generated. The imprinting effect of the iQTL explained 2% of the phenotypic variance in the U-imF_2_ or R-imF_2_ population. Six different types of iQTL in terms of the effects (*a*, *d*, and *i*) were investigated, including the full-effect type, in which all sorts of effect exist, and five partial-effect types, in which either additive effect or dominance effect, or both do not exist (Table 2; Cheverud et al., 2008). The simulated data were analyzed with PM-1 and PM-2, respectively. Each case was simulated for 500 times. LOD thresholds for PM-1 and PM-2 at the overall significance level of 0.05 were estimated by simulation under the null hypothesis with 5,000 replicates. The results (Table 2) showed:

**TABLE 2:** Simulation results of point mapping of a single iQTL.

**Type^a^**	**Design**	**Method**	**Pos. (cM)^b^**	***a***	***d***	***i***	**Power (%)^c^**
FULL	U-imF_2_	PM-1	49.65 ± 9.01	1.80 ± 0.60	1.60 ± 0.86	2.22 ± 0.52	92.2
(0.05)		PM-2	49.91 ± 2.86	2.04 ± 0.51	2.10 ± 0.74	2.15 ± 0.41	86.6 (99.6)
	R-imF_2_	PM-1	50.27 ± 7.71	1.80 ± 0.57	1.66 ± 0.81	2.15 ± 0.48	92.8
		PM-2	49.96 ± 3.25	1.99 ± 0.51	2.00 ± 0.76	2.11 ± 0.40	88.2 (99.8)
		Real value	50	2	2	2	
DIPOD	U-imF_2_	PM-1	49.98 ± 7.32	−0.03 ± 0.53	1.57 ± 0.79	2.21 ± 0.41	90.8
(0.03)		PM-2	49.90 ± 3.49	−0.03 ± 0.57	2.01 ± 0.71	2.17 ± 0.42	82.6 (95.0)
	R-imF_2_	PM-1	49.88 ± 7.03	0.01 ± 0.53	1.70 ± 0.81	2.16 ± 0.43	91.8
		PM-2	49.76 ± 4.40	−0.01 ± 0.56	2.09 ± 0.76	2.14 ± 0.40	85.8 (92.2)
		Real value	50	0	2	2	
DIPUD	U-imF_2_	PM-1	49.86 ± 6.42	−0.04 ± 0.51	1.68 ± 0.81	−2.21 ± 0.50	89.8
(0.03)		PM-2	49.58 ± 3.79	−0.04 ± 0.56	2.04 ± 0.75	−2.21 ± 0.43	82.4 (92.8)
	R-imF_2_	PM-1	50.02 ± 7.10	0.03 ± 0.48	1.66 ± 0.82	−2.19 ± 0.43	91.6
		PM-2	50.44 ± 5.15	0.04 ± 0.53	1.99 ± 0.79	−2.17 ± 0.40	87.4 (93.0)
		Real value	50	0	2	−2	
PEP	U-imF_2_	PM-1	50.43 ± 9.80	1.77 ± 0.57	−0.02 ± 0.72	2.22 ± 0.46	91.6
(0.04)		PM-2	50.02 ± 4.46	2.03 ± 0.53	−0.01 ± 0.81	2.17 ± 0.43	86.4 (99.0)
	R-imF_2_	PM-1	50.57 ± 7.43	1.83 ± 0.58	0.01 ± 0.72	2.21 ± 0.41	92.4
		PM-2	50.01 ± 4.43	2.02 ± 0.54	0.02 ± 0.82	2.16 ± 0.43	88.8 (97.8)
		Real value	50	2	0	2	
PEM	U-imF_2_	PM-1	49.69 ± 8.61	1.79 ± 0.54	−0.06 ± 0.74	−2.23 ± 0.40	90.2
(0.04)		PM-2	49.90 ± 5.17	2.00 ± 0.53	−0.05 ± 0.82	−2.16 ± 0.43	87.8 (99.2)
	R-imF_2_	PM-1	49.69 ± 7.45	1.79 ± 0.56	−0.03 ± 0.71	−2.18 ± 0.44	91.2
		PM-2	49.94 ± 4.76	2.02 ± 0.54	−0.03 ± 0.81	−2.13 ± 0.42	89.0 (98.6)
		Real value	50	2	0	−2	
DIB	U-imF_2_	PM-1	50.07 ± 7.87	0.01 ± 0.50	0.07 ± 0.70	2.23 ± 0.45	89.0
(0.02)		PM-2	50.05 ± 7.32	0.01 ± 0.59	0.05 ± 0.87	2.33 ± 0.38	71.0 (76.4)
	R-imF_2_	PM-1	49.61 ± 8.22	−0.02 ± 0.51	0.02 ± 0.70	2.17 ± 0.45	89.2
		PM-2	49.70 ± 7.42	−0.02 ± 0.59	0.00 ± 0.91	2.27 ± 0.43	69.6 (71.0)
		Real value	50	0	0	2	

***^a^**FULL, full-effect type (a = d = i); DIPOD, dominance imprinting, polar, over dominance (a = 0∩d = i); DIPUD, dominance imprinting, polar, under dominance (a = 0∩d = −i); PEP, parental expression, paternal (a = i∩d = 0); PEM, parental expression, maternal (a = −i∩d = 0); DIB, dominance imprinting, bipolar (a = 0∩d = 0). The data in parenthesis are the total proportion of phenotypic variance explained by the iQTL (the proportion of phenotypic variance explained by the imprinting effect of the iQTL is 0.02 in each type). ^b^The estimates of position and effects are shown as “mean ± standard deviation.” ^c^The powers were estimated based on 500 times of simulation. The data in parenthesis are the power of QTL detection in PM-2 (step 1). The LOD thresholds for PM-1, step 1 of PM-2, and step 2 of PM-2 were 1.97, 3.25, and 1.79 in U-imF_2_ and 1.97, 3.36, and 1.80 in R-imF_2_, respectively.*

(i) When other conditions (iQTL type and mapping method) were fixed, the results (means and standard deviations of estimates and statistical powers) obtained under the two designs were all very similar, suggesting that the two designs are basically equivalent for iQTL mapping.
(ii) Except in the case of type DIB, which had no additive and dominance effects, the power of QTL detection in PM-2 (step 1) was always higher than the power of iQTL detection in PM-1, and the difference was especially large in the cases of type FULL, PEP and PEM. However, the power of iQTL detection in PM-2 (step 2) was always lower than that in PM-1, and the difference was especially large in the case of type DIB.
(iii) The mean estimates of iQTL position obtained by PM-1 and PM-2 were very close to the real value in all the cases, suggesting that a single iQTL can be unbiasedly mapped by both methods. However, the standard deviation of iQTL position obtained by PM-2 was always significantly smaller than that obtained by PM-1 except in the case of type DIB. Even for type DIB, the former was still a little smaller than the latter. This suggests that PM-2 is more precise than PM-1 for iQTL mapping in general.
(iv) The estimation results of imprinting effect obtained by PM-1 and PM-2 were similar in all the cases. Noticeably, the means were always a little larger than the real value, suggesting that both methods may slightly overestimate the imprinting effect. For the additive and dominance effects, the means obtained by PM-2 were very close to the real values, suggesting that the estimation is unbiased; but the means obtained by PM-1 were always obviously smaller than the real values, suggesting that PM-1 may underestimate the additive and dominance effects. These results suggest that PM-2 is better than PM-1 for estimating the additive and dominance effects of iQTL.

### Simulation Study II

**FIGURE 1:**
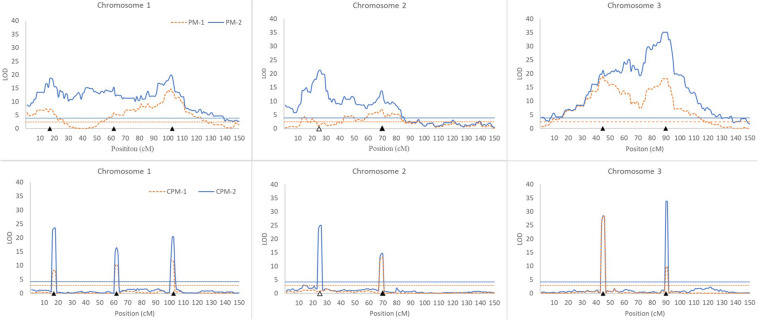
Results of QTL scanning by PM methods **(upper)** and CPM methods **(lower)** in an assumed genome consisting of three chromosomes. The horizontal lines indicate LOD thresholds at the overall significance level of 0.05. The filled and blank triangles indicate the positions of iQTL and niQTL, respectively.

In this simulation study, we assumed that a species had three pairs of chromosomes, each of which was 150 cM in length and had one marker every cM. There were seven QTLs in the genome, including three iQTLs on chromosome 1, one iQTLs and one niQTL on chromosome 2, and two iQTLs on chromosome 3 (Table 3). An R-imF_2_ population comprising 800 hybrid lines was generated from a DH population of 100 lines. Each QTL accounted for ∼7% of the phenotypic variance in the R-imF_2_ population. The simulated data were analyzed with PM-1, PM-2, CPM-1, and CPM-2, respectively. Cofactors for CPM-1 and CPM-2 were selected by stepwise regression at the significance level of 0.05. LOD thresholds at the overall significance level of 0.05 were estimated by permutation test with 1,000 replicates. The results (Figure 1 and Table 3) showed:

**TABLE 3:** Simulation results of mapping multiple iQTLs using PM-1, PM-2, CPM-1, and CPM-2.

**Chr.**	**QTL^a^**	**Type**	**Method**	**Pos. (cM)^b^**	***a*^b^**	***d*^b^**	***i*^b^**
1	Q1-1	PEP	PM-1	17	1.23	0.28	0.93
	(7.06, 3.53)		PM-2	17	1.23	0.28	0.93
			CPM-1	17	0.76	0.13	0.90
			CPM-2	17	0.76	0.13	0.90
			Real value	17	0.84	0	0.84
	Q1-2	DIPUD	PM-1	(62)	(0.24)	(1.07)	(−0.85)
	(7.06, 4.70)		PM-2	(62)	(0.24)	(1.07)	(−0.85)
			CPM-1	62	−0.09	086	−1.01
			CPM-2	62	−0.09	0.86	−1.01
			Real value	62	0	0.97	−0.97
	Q1-3	PEM	PM-1	102	0.86	0.08	−1.29
	(7.06, 3.53)		PM-2	102	0.86	0.08	−1.29
			CPM-1	102	0.77	0.02	−0.89
			CPM-2	103	0.79	0.03	−0.86
			Real value	103	0.84	0	−0.84
2	Q2-1	Non-imprinted	PM−1	ns	ns	ns	ns
	(7.06, 0)		PM-2	25	1.42	1.30	0.37
			CPM-1	ns	ns	ns	ns
			CPM-2	25	1.03	1.17	0.03
			Real value	25	0.97	0.97	0
	Q2-2	DIPOD	PM-1	70	0.38	0.90	0.96
	(7.06, 4.70)		PM-2	70	0.38	0.90	0.96
			CPM-1	70	−0.01	0.89	0.94
			CPM-2	70	−0.01	0.89	0.94
			Real value	70	0	0.97	0.97
3	Q3-1	DIB	PM-1	45	0.46	0.30	1.56
	(6.96, 6.96)		PM-2	(45)	(0.46)	(0.30)	(1.56)
			CPM-1	45	−0.08	0.08	1.29
			CPM-2	45	−0.08	0.08	1.29
			Real value	45	0	0	1.18
	Q3-2	FULL	PM-1	90	1.15	0.97	1.48
	(7.03, 2.81)		PM-2	90	1.15	0.97	1.48
			CPM-1	90	0.83	0.79	0.81
			CPM-2	90	0.83	0.79	0.81
			Real value	90	0.75	0.75	0.75

*^*a*^The data in parenthesis are the percentages of phenotypic variance explained by the QTL and its imprinting effect, respectively. ^*b*^The estimates in parenthesis were obtained based on unremarkable or unclear LOD peaks. ns, not significant. The LOD thresholds for PM-1, step 1 of PM-2, step 2 of PM-2, CPM-1, step 1 of CPM-2, and step 2 of CPM-2 were 2.42, 3.82, 2.37, 2.85, 4.15, and 2.79, respectively.*

(i) All of the iQTLs were precisely mapped by both CPM-1 and CPM-2, and the estimates of iQTL positions obtained by the two methods were almost completely the same (with only a slight difference at Q1-3). PM-1 and PM-2 also precisely mapped some of the iQTLs. These two methods obtained exactly the same estimates of iQTL positions. However, the LOD peaks of PM-1 and PM-2 were broad. In addition, there were many small peaks, which may make it difficult to identify the peaks of true iQTLs (such as the peaks of Q1-2 in PM-1 and PM-2, and the peak of Q3-1 in PM-2) and result in ghost or false iQTLs (such as the peak on the left of Q2-1 and that between Q2-1 and Q2-2 in PM-1, and the peaks between Q1-1 and Q1-2, between Q2-1 and Q2-2, and between Q3-1 and Q3-1 in PM-2).
(ii) Corresponding to the estimation of iQTL positions, the estimates of iQTL effects were also completely the same between PM-1 and PM-2 and almost completely the same between CPM-1 and CPM-2, respectively. In most of the cases, the estimates of effects obtained by CPM-1 and CPM-2 were closer to the real values than those obtained by PM-1 and PM-2.
(iii) The LOD peaks of PM-2 were always higher than those of PM-1. This is consistent with the results of simulation study I. For the same reason, the LOD peaks of CPM-2 were higher than those of CPM-1 except for the DIB-type iQTL (Q3-1). In addition, as expected, the niQTL (Q2-1) was mapped only by PM-2 and CPM-2, respectively.

## Discussion

The advantages of iQTL mapping based on imF_2_ populations have been demonstrated before (Wen and Wu, 2014). R-imF_2_ is a special type of imF_2_ population. Although the simulation study results suggest that R-imF_2_ does not apparently improve the result of iQTL mapping in comparison with U-imF_2_ (Table 2), it is expected to be advantageous for experimental operation. For each cross combination, only one hybrid line is produced in U-imF_2_, while two hybrid lines are produced in R-imF_2_. Therefore, R-imF_2_ only needs half of the number of combinations used in U-imF_2_. This makes the cross work more convenient and may, to some extent, alleviate the workload of developing the imF_2_ population.

According to the simulation study results, PM-2 can estimate the position and the additive and dominance effects of an iQTL more precisely than PM-1 (Table 2). This is understandable. PM-1 estimates the position of an iQTL based on its imprinting effect only, while PM-2 estimates the position of an iQTL based on not only its imprinting effect, but also its additive and dominance effects. Obviously, the latter would have a higher statistical power as long as the additive and dominance effects exist (Table 2). This would surely increase the estimation precision of iQTL position and therefore increase the estimation precision of iQTL effects.

Although PM-2 can noticeably improve the estimation of iQTL position and effects, the power of detecting iQTL in PM-2 is always lower than that in PM-1 (Table 2). This means that there is a cost of losing power for gaining precision in PM-2. The reason may be that an iQTL detected by PM-1 is at the position, where the imprinting effect has the highest significance, while the iQTL position estimated by PM-2 may not be at the point, where the imprinting effect is the most significant. Nevertheless, the power difference of iQTL detection between PM-1 and PM-2 is not large except in the case of type DIB (Table 2). Therefore, the improvement of estimation precision achieved by PM-2 is cost worthy.

Although the PM methods behave well in the mapping of a single iQTL, they are not ideal for multiple iQTL mapping (Figure 1). In practice, therefore, it is more appropriate to use the CPM methods. PM-2 demonstrates the merit of two-step analysis. CPM-2 also exhibits the merit of higher LOD score in the identification of QTL position (Figure 1). However, the LOD peaks obtained by CPM-1 and CPM-2 usually have the same width for the same iQTL (Figure 1). This suggests that the two methods have similar resolution in iQTL mapping. Therefore, the higher LOD score of CPM-2 might have little help for increasing the precision of iQTL mapping, probably due to the role of cofactors. Nevertheless, CPM-2 still has an advantage over CPM-1, namely, it can map both iQTLs and niQTLs.

Considering that the basic principle and conclusions of iQTL mapping may not change with the density of markers (Wen and Wu, 2014), we did not consider in this paper the situation of iQTL mapping based on conventional low-density maps. The methods described above can be directly applied to the conventional map as long as the values of the dummy variables at the position to be tested in Eqs. (1 and 4) are replaced with the expected values calculated according to the flanking markers (Wen and Wu, 2014).

Power is the most frequently used index for evaluating the efficiency of a QTL mapping method, which can reflect the probability of type II error. Besides, false discovery rate (FDR) may be also an important index for the evaluation because it can reflect the probability of type I error (Li et al., 2012b). A good QTL mapping method should have higher power but lower FDR. Similar to the power, the FDR in QTL mapping can also be estimated by computer simulation (Li et al., 2012b). In the simulation study I of this study, one QTL was set at the center of a chromosome of 50 cM in length in each case. Since the QTL really existed, a single QTL detected on the chromosome could be always regarded as true, although the estimated QTL position was very imprecise (far from the real position) sometimes. Certainly, if there were two or more QTLs detected on the chromosome simultaneously, the additional QTL should be false. However, such situations did not occur in the simulations. Therefore, the FDR was always zero in our simulation study. In other words, the conditions assumed in our simulation study avoided the occurrence of false discovery. This was beneficial to the comparative study based on the power.

In this study, we only consider the iQTL mapping based on one-environment experiment. However, the genetic model can be easily extended to adapt the data of multi-environment experiment, from which the QTL-by-environment interaction can be analyzed using suitable statistical methods such as the mixed linear model approach, which has been used for mapping QTLs with the digenic epistasis and QTL-by-environment interaction as well as additive and dominance effects based on imF_2_ population (Gao and Zhu, 2007).

## Data Availability Statement

All datasets generated for this study are included in the article/supplementary material, further inquiries can be directed to the corresponding authors.

## Author Contributions

All authors listed have made a substantial, direct and intellectual contribution to the work, and approved it for publication.

## Conflict of Interest

The authors declare that the research was conducted in the absence of any commercial or financial relationships that could be construed as a potential conflict of interest.

## Footnotes

1 The software package of our methods is available by contacting us through email.

## References

[B1] Babak T. Deveale B. Armour C. Raymond C. Cleary M. A. van der Kooy D. (2008). Global survey of genomic imprinting by transcriptome sequencing. *Curr. Biol.* 18 1735–1741. 10.1016/j.cub.2008.09.044 19026546

[B2] Bauer M. J. Fischer R. L. (2011). Genome demethylation and imprinting in the endosperm. *Curr. Opin. Plant Biol.* 14 162–167. 10.1016/j.pbi.2011.02.006 21435940 PMC3082360

[B3] Cheverud J. M. Hager R. Roseman C. Fawcett G. Wang B. Wolf J. B. (2008). Genomic imprinting effects on adult body composition in mice. *Proc. Natl. Acad. Sci. U.S.A.* 105 4253–4258. 10.1073/pnas.0706562105 18337500 PMC2393747

[B4] Churchill G. A. Doerge R. W. (1994). Empirical threshold values for quantitative trait mapping. *Genetics* 138 963–971. 7851788 10.1093/genetics/138.3.963 PMC1206241

[B5] Croteau A. K. Croteau N. (2004). Mechanisms of epigenetic variation: polymorphic imprinting. *Curr. Genomics* 5 417–429.

[B6] Cui Y. (2007). A statistical framework for genome-wide scanning and testing of imprinted quantitative trait loci. *J. Theor. Biol.* 244 115–126. 10.1016/j.jtbi.2006.07.009 16959270

[B7] Cui Y. Cheverud J. M. Wu R. (2007). A statistical model for dissecting genomic imprinting through genetic mapping. *Genetica* 130 227–239. 10.1007/s10709-006-9101-x 16955328

[B8] Cui Y. Li S. Li G. (2008). Functional mapping imprinted quantitative trait loci underlying developmental characteristics. *Theor. Biol. Med. Modelling* 5:6. 10.1186/1742-4682-5-6 18346281 PMC2324076

[B9] Cui Y. Lu Q. Cheverud J. M. Littell R. C. Wu R. (2006). Model for mapping imprinted quantitative trait loci in an inbred F2 design. *Genomics* 87 543–551. 10.1016/j.ygeno.2005.11.021 16413163

[B10] Danilevskaya O. N. Hermon P. Hantke S. Muszynski M. G. Kollipara K. Ananiev E. V. (2003). Duplicated fie genes in maize: expression pattern and imprinting suggest distinct functions. *Plant Cell* 15 425–438. 10.1105/tpc.006759 12566582 PMC141211

[B11] de Koning D. J. Rattink A. P. Harlizius B. van Arendonk J. A. Brascamp E. W. Groenen M. A. (2000). Genome-wide scan for body composition in pigs reveals important role of imprinting. *Proc. Natl. Acad. Sci. U.S.A.* 97 7947–7950. 10.1073/pnas.140216397 10859367 PMC16650

[B12] Gao Y. M. Zhu J. (2007). Mapping QTLs with digenic epistasis under multiple environments and predicting heterosis based on QTL effects. *Theor. Appl. Genet.* 115 325–333. 17534594 10.1007/s00122-007-0564-7

[B13] Girardot M. Feil R. Llères D. (2013). Epigenetic deregulation of genomic imprinting in humans: causal mechanisms and clinical implications. *Epigenomics* 5 715–728. 10.2217/epi.13.66 24283884

[B14] Hagan J. P. O’Neill B. L. Stewart C. L. Kozlov S. V. Croce C. M. (2009). At least ten genes define the imprinted Dlk1-Dio3 cluster on mouse chromosome 12qF1. *PLoS One* 4:e4352. 10.1371/journal.pone.0004352 19194500 PMC2632752

[B15] Hager R. Cheverud J. M. Wolf J. B. (2008). Maternal effects as the cause of parent-of-origin effects that mimic genomic imprinting. *Genetics* 178 1755–1762. 10.1534/genetics.107.080697 18245362 PMC2278069

[B16] Haghighi F. Hodge S. E. (2002). Likelihood formulation of parent-of-origin effects on segregation analysis, including ascertainment. *Am. J. Hum. Genet.* 70 142–156. 10.1086/324709 11741195 PMC384884

[B17] Haley C. S. Knott S. A. Elsen J. M. (1994). Mapping quantitative trait loci in crosses between outbred lines using least squares. *Genetics* 136 1195–1207. 8005424 10.1093/genetics/136.3.1195 PMC1205874

[B18] Hanson R. L. Kobes S. Lindsay R. S. Knowler W. C. (2001). Assessment of parent-of-origin effects in linkage analysis of quantitative traits. *Am. J. Hum. Genet.* 68 951–962. 10.1086/319508 11254452 PMC1275649

[B19] Haun W. J. Laoueillé-Duprat S. O’connell M. J. Spillane C. Grossniklaus U. Phillips A. R. (2007). Genomic imprinting, methylation and molecular evolution of maize Enhancer of zeste (Mez) homologs. *Plant J.* 49 325–337. 17181776 10.1111/j.1365-313X.2006.02965.x

[B20] Hur S. K. Freschi A. Ideraabdullah F. Thorvaldsen J. L. Luense L. J. Weller A. H. (2016). Humanized H19/Igf2 locus reveals diverged imprinting mechanism between mouse and human and reflects silver-russell syndrome phenotypes. *Proc. Natl. Acad. Sci. U.S.A.* 113 10938–10943. 10.1073/pnas.1603066113 27621468 PMC5047210

[B21] Ikeda Y. (2012). Plant imprinted genes identified by genome-wide approaches and their regulatory mechanisms. *Plant Cell Physiol.* 53 809–816. 10.1093/pcp/pcs049 22492232

[B22] Jiang J. Shen B. O’Connell J. R. VanRaden P. M. Cole J. B. Ma L. (2017). Dissection of additive, dominance, and imprinting effects for production and reproduction traits in Holstein cattle. *BMC Genomics* 18:425. 10.1186/s12864-017-3821-4 28558656 PMC5450346

[B23] Karami K. Zerehdaran S. Javadmanesh A. Shariati M. M. Fallahi H. (2019). Characterization of bovine (*Bos taurus*) imprinted genes from genomic to amino acid attributes by data mining approaches. *PLoS One* 14:e0217813. 10.1371/journal.pone.0217813 31170205 PMC6553745

[B24] Kermicle J. L. (1970). Dependence of the R-mottled aleurone phenotype in maize on mode of sexual transmission. *Genetics* 66 69–85. 17248508 10.1093/genetics/66.1.69 PMC1212486

[B25] Knapp M. Strauch K. (2004). Affected-sib-pair test for linkage based on constraints for identical-by-descent distributions corresponding to disease models with imprinting. *Genet. Epidemiol.* 26 273–285. 10.1002/gepi.10320 15095387

[B26] Knott S. A. Marklund L. Haley C. S. Andersson K. Davies W. Ellegren H. (1998). Multiple marker mapping of quantitative trait loci in a cross between outbred wild boar and large white pigs. *Genetics* 149 1069–1080. 9611214 10.1093/genetics/149.2.1069 PMC1460207

[B27] Lawson H. A. Cheverud J. M. Wolf J. B. (2013). Genomic imprinting and parent-of-origin effects on complex traits. *Nat. Rev. Genet.* 14 609–617. 10.1038/nrg3543 23917626 PMC3926806

[B28] Li H. H. Zhang L. Y. Wang J. K. (2012b). Estimation of statistical power and false discovery rate of QTL mapping methods through computer simulation. *Chin. Sci. Bull.* 57:27012710 10.1007/s11434-012-5239-3

[B29] Li S. Wang X. Li J. Yang T. Min L. Liu Y. (2012a). Bayesian mapping of genome-wide epistatic imprinted loci for quantitative traits. *Theor. Appl. Genet.* 124 1561–1571. 10.1007/s00122-012-1810-1 22350088

[B30] Li Y. Coelho C. M. Liu T. Wu S. Wu J. Zeng Y. (2008). A statistical model for estimating maternal-zygotic interactions and parent-of-origin effects of QTLs for seed development. *PLoS One* 3:e3131. 10.1371/journal.pone.0003131 18769549 PMC2519836

[B31] Liu T. Todhunter R. J. Wu S. Hou W. Mateescu R. Zhang Z. (2007). A random model for mapping imprinted quantitative trait loci in a structured pedigree: an implication for mapping canine hip dysplasia. *Genomics* 90 276–284. 10.1016/j.ygeno.2007.04.004 17531439

[B32] Long J. E. Cai X. (2007). Igf-2r expression regulated by epigenetic modification and the locus of gene imprinting disrupted in cloned cattle. *Gene* 388 125–134. 10.1016/j.gene.2006.10.014 17150312

[B33] Luo M. Platten D. Chaudhury A. Peacock W. J. Dennis E. S. (2009). Expression, imprinting, and evolution of rice homologs of the polycomb group genes. *Mol. Plant* 2 711–723. 10.1093/mp/ssp036 19825651

[B34] Mackay D. Temple I. K. (2017). Human imprinting disorders: principles, practice, problems and progress. *Eur. J. Med. Genet.* 60 618–626. 10.1016/j.ejmg.2017.08.014 28818477

[B35] Mantey C. Brockmann G. A. Kalm E. Reinsch N. (2005). Mapping and exclusion mapping of genomic imprinting effects in mouse F2 families. *J. Hered.* 96 329–338. 10.1093/jhered/esi044 15761081

[B36] Morison I. M. Ramsay J. P. Spencer H. G. (2005). A census of mammalian imprinting. *Trends Genet.* 21 457–465. 10.1016/j.tig.2005.06.008 15990197

[B37] Nolan C. M. Killian J. K. Petitte J. N. Jirtle R. L. (2001). Imprint status of M6P/IGF2R and IGF2 in chickens. *Dev. Genes Evol.* 211 179–183. 10.1007/s004270000132 11455432

[B38] Pei L. Zhang L. Li J. Shen C. Qiu P. Tu L. (2019). Tracing the origin and evolution history of methylation-related genes in plants. *BMC Plant Biol.* 19:307. 10.1186/s12870-019-1923-7 31299897 PMC6624907

[B39] Pembrey M. Saffery R. Bygren L. O. Network in epigenetic epidemiology, and Network in epigenetic epidemiology (2014). Human transgenerational responses to early-life experience: potential impact on development, health and biomedical research. *J. Med. Genet.* 51 563–572. 10.1136/jmedgenet-2014-102577 25062846 PMC4157403

[B40] Pratt S. C. Daly M. J. Kruglyak L. (2000). Exact multipoint quantitative-trait linkage analysis in pedigrees by variance components. *Am. J. Hum. Genet.* 66 1153–1157. 10.1086/302830 10712227 PMC1288151

[B41] Raissig M. T. Baroux C. Grossniklaus U. (2011). Regulation and flexibility of genomic imprinting during seed development. *Plant Cell* 23 16–26. 10.1105/tpc.110.081018 21278124 PMC3051244

[B42] Sandovici I. Kassovska-Bratinova S. Loredo-Osti J. C. Leppert M. Suarez A. Stewart R. (2005). Interindividual variability and parent of origin DNA methylation differences at specific human Alu elements. *Hum. Mol. Genet.* 14 2135–2143. 10.1093/hmg/ddi218 15972727

[B43] Santure A. W. Spencer H. G. (2011). Quantitative genetics of genomic imprinting: a comparison of simple variance derivations, the effects of inbreeding, and response to selection. *G3* 1 131–142. 10.1534/g3.111.000042 22384325 PMC3276129

[B44] Shete S. Amos C. I. (2002). Testing for genetic linkage in families by a variance-components approach in the presence of genomic imprinting. *Am. J. Hum. Genet.* 70 751–757. 10.1086/338931 11836650 PMC384951

[B45] Shete S. Zhou X. Amos C. I. (2003). Genomic imprinting and linkage test for quantitative-trait Loci in extended pedigrees. *Am. J. Hum. Genet.* 73 933–938. 10.1086/378592 13680523 PMC1180613

[B46] Spencer H. G. (2002). The correlation between relatives on the supposition of genomic imprinting. *Genetics* 161 411–417. 12019254 10.1093/genetics/161.1.411 PMC1462108

[B47] Strauch K. Fimmers R. Kurz T. Deichmann K. A. Wienker T. F. Baur M. P. (2000). Parametric and nonparametric multipoint linkage analysis with imprinting and two-locus-trait models: application to mite sensitization. *Am. J. Hum. Genet.* 66 1945–1957. 10.1086/302911 10796874 PMC1378058

[B48] Wang C. Wang Z. Prows D. R. Wu R. (2012). A computational framework for the inheritance pattern of genomic imprinting for complex traits. *Brief. Bioinform.* 13 34–45. 10.1093/bib/bbr023 21565936 PMC3278998

[B49] Wen Y. Wu W. (2014). Mapping of imprinted quantitative trait loci using immortalized F2 populations. *PLoS One* 9:e92989. 10.1371/journal.pone.0092989 24676330 PMC3968037

[B50] Wolf J. B. Hager R. Cheverud J. M. (2008). Genomic imprinting effects on complex traits: a phenotype-based perspective. *Epigenetics* 3 295–299. 10.4161/epi.3.6.7257 19029803

[B51] Wu R. L. Ma C. X. Wu S. S. Zeng Z. B. (2002). Linkage mapping of sex-specific differences. *Genet Res.* 79 85–96. 11974606 10.1017/s0016672301005389

[B52] Yang R. Wang X. Wu Z. Prows D. R. Lin M. (2010). Bayesian model selection for characterizing genomic imprinting effects and patterns. *Bioinformatics* 26 235–241. 10.1093/bioinformatics/btp620 19880366 PMC2804294

[B53] Zeng Z. B. (1994). Precision mapping of quantitative trait loci. *Genetics* 136 1457–1468. 8013918 10.1093/genetics/136.4.1457 PMC1205924

[B54] Zhang M. Zhao H. Xie S. Chen J. Xu Y. Wang K. (2011). Extensive, clustered parental imprinting of protein-coding and noncoding RNAs in developing maize endosperm. *Proc. Natl. Acad. Sci. U.S.A.* 108 20042–20047. 10.1073/pnas.1112186108 22114195 PMC3250141

[B55] Zhou X. Fang M. Li J. Prows D. R. Yang R. (2012). Characterization of genomic imprinting effects and patterns with parametric accelerated failure time model. *Mol. Genet. Genomics* 287 67–75. 10.1007/s00438-011-0661-9 22143178

